# Beyond viral suppression: combining PEG-interferon with novel immunotherapies for functional cure of chronic hepatitis B

**DOI:** 10.3389/fcimb.2026.1779323

**Published:** 2026-06-04

**Authors:** Qi Wang, Wen Deng, Shiyu Wang, Weihua Cao, Xinxin Li, Ziyu Zhang, Yao Xie, Huichun Xing, Minghui Li

**Affiliations:** 1 Department of Hepatology Division 2, Beijing Ditan Hospital, Capital Medical University, Beijing, China; 2 HBV Infection, Clinical Cure and Immunology Joint Laboratory for Clinical Medicine, Capital Medical University, Beijing, China; 3 Department of Hepatology Division 2, Peking University Ditan Teaching Hospital, Beijing, China

**Keywords:** chronic hepatitis B, functional cure, HBsAg loss, immune reprogramming, PEG-interferon

## Abstract

Chronic hepatitis B (CHB) affects approximately 250 million people worldwide. The major barrier to cure lies in the persistent presence of covalently closed circular DNA (cccDNA) and integrated HBV DNA within hepatocytes, which continuously drive hepatitis B surface antigen (HBsAg) expression and maintain immune tolerance, thereby leading to functional exhaustion of antiviral effector cells. Although nucleos(t)ide analogues (NAs) effectively suppress viral replication, they have limited impact on cccDNA activity and antigen production. In contrast, pegylated interferon-α (PEG-IFN-α) enhances antigen presentation, activates innate immunity, and partially restores HBV-specific T cell function, thereby contributing to the disruption of immune tolerance to some extent. However, its therapeutic efficacy remains influenced by host immune status and antigen burden. With the development of antigen-reduction strategies (such as siRNA/antisense oligonucleotides [ASO] and HBsAg-targeting monoclonal antibodies), therapeutic vaccines, and immune-modulatory approaches, PEG-IFN-α is increasingly being incorporated into combination therapies. This review summarizes its immunological basis and clinical advances, and further discusses biomarker-driven patient stratification strategies, with the aim of improving functional cure rates in CHB.

## 1 Introduction

Chronic hepatitis B (CHB) remains a major global public health concern, with approximately 250 million people chronically infected with hepatitis B virus (HBV), and about 1.1 million deaths each year due to decompensated cirrhosis and hepatocellular carcinoma (Jeng et al., 2023; Marrapu and Kumar, 2024; European Association for the Study of the L, 2025).The persistence of HBV is primarily attributed to covalently closed circular DNA (cccDNA) and HBV DNA integrated into the host genome (Xia and Guo, 2020; Allweiss et al., 2023), which support sustained viral protein transcription, particularly hepatitis B surface antigen (HBsAg), thereby maintaining immune tolerance and limiting effective immune-mediated clearance of infected hepatocytes (Nassal, 2015; Alter et al., 2018; Huang et al., 2023).

During HBV infection, following entry into hepatocytes, relaxed circular DNA (rcDNA) is transported into the nucleus and converted into cccDNA (Boonstra and Sari, 2025), which serves as the transcriptional template for multiple viral RNAs, including precore RNA, pregenomic RNA (pgRNA), surface protein mRNAs, and X mRNA. Among these, pgRNA plays a key role in viral replication, which is completed through reverse transcription within the nucleocapsid, generating new viral DNA (Tsukuda and Watashi, 2020). Following replication, a portion of nucleocapsids recycles back to the nucleus to replenish the cccDNA pool, while the remainder undergoes envelopment and is secreted as mature virions. Notably, infected hepatocytes secrete large excesses of non-infectious subviral particles (SVPs) composed mainly of HBsAg; in chronic HBV infection, only approximately one in 10,000 circulating viral particles is infectious, with non-infectious particles accounting for the vast majority in serum (Mohebbi et al., 2018). These particles lack viral genomic material but are abundantly present in circulation and represent a major source of serum HBsAg.

Persistent antigen expression constitutes a central feature of immune dysregulation in CHB. HBsAg and hepatitis B e antigen (HBeAg) are continuously present during HBV infection. HBsAg is primarily translated from surface protein mRNAs and can also be derived from transcripts of integrated HBV DNA, whereas HBeAg is translated from precore RNA and subsequently processed and secreted. HBeAg is commonly used as a marker reflecting viral replication activity and host immune status. On this basis, chronic HBV infection is classically divided into four clinical phases: the immune-tolerant phase, immune-active (immune clearance) phase, inactive carrier phase, and HBeAg-negative reactivation phase, which differ in viral replication levels, antigen burden, and host immune responses.

The current therapeutic goal is to achieve a functional cure, defined as sustained clearance of HBsAg with or without the development of anti-HBs, which is associated with a significantly reduced risk of cirrhosis and hepatocellular carcinoma. Nucleos(t)ide analogues (NAs) (Sadler and Williams, 2008; Ho et al., 2024) effectively suppress HBV replication and maintain long-term undetectable HBV DNA levels; however, their impact on cccDNA activity and HBsAg production is limited, and the rate of HBsAg clearance remains low during prolonged treatment (Zeisel et al., 2015; Wong et al., 2022).In contrast, pegylated interferon-α (PEG-IFN-α) exerts its effects by modulating both innate and adaptive immunity and can induce HBsAg decline or even clearance in a subset of patients. Its efficacy is closely associated with host immune status and antigen burden (Zoulim et al., 2016; You et al., 2023).

**Figure 1:**
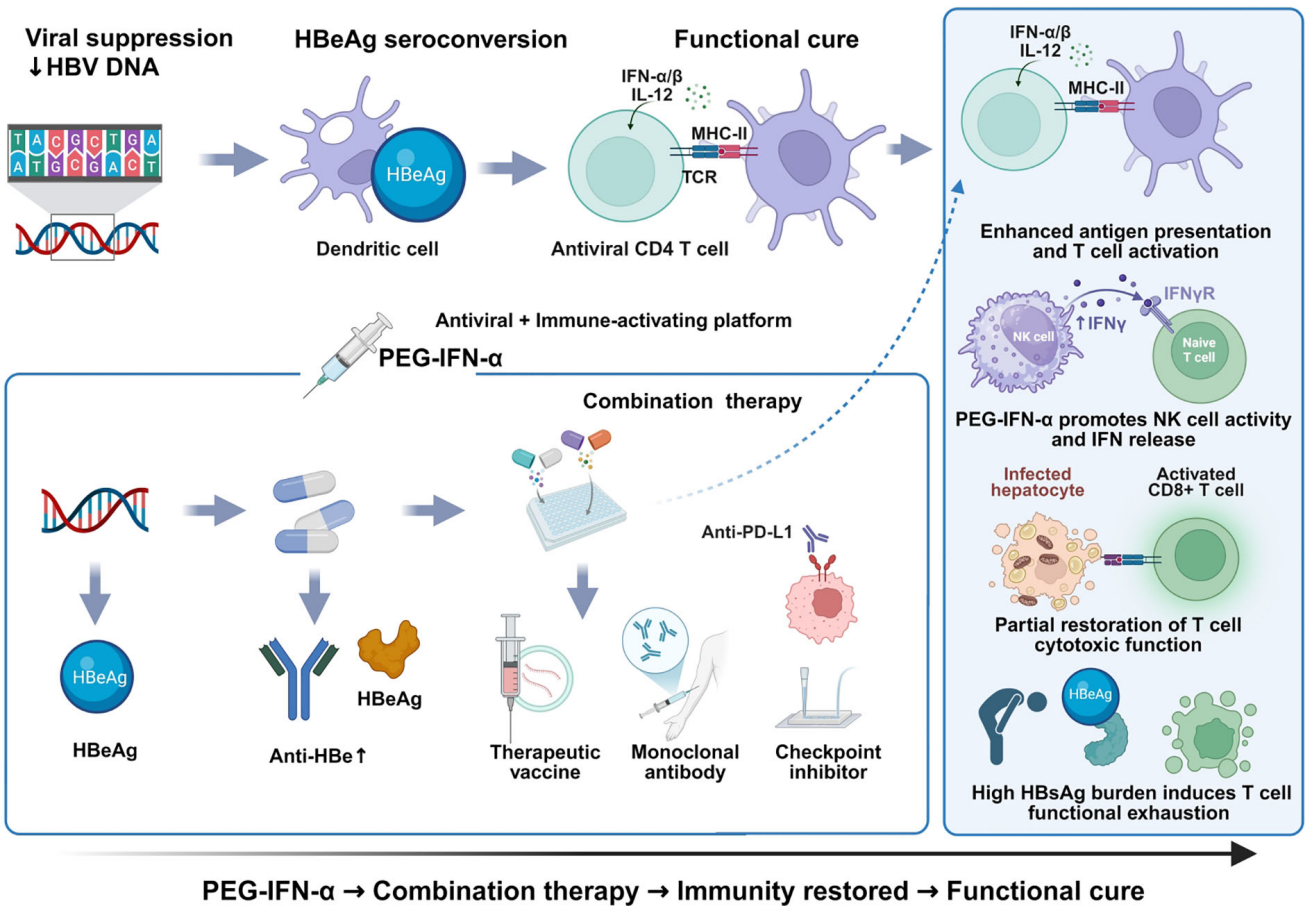
Schematic illustration of PEG-IFN-α–based strategies for functional cure in CHB. The upper panel illustrates the conceptual progression of CHB treatment from virological suppression toward functional cure. From left to right, the stages represent reduction of HBV DNA, HBeAg seroconversion, and functional cure characterized by HBsAg loss. During this process, dendritic cells mediate antigen uptake and presentation, and interactions between the TCR and MHC-II molecules promote antiviral CD4^+^ T-cell responses. The lower panel summarizes treatment strategies. On the basis of viral suppression achieved by NAs, PEG-IFN-α serves as an antiviral and immune-activating platform and may be combined with therapeutic vaccines, monoclonal antibodies, and immune checkpoint inhibitors. The right panel depicts representative immune processes associated with PEG-IFN-α treatment, including enhanced antigen presentation, NK-cell activation, IFN-γ release, restoration of CD8^+^ T-cell cytotoxicity, and partial reversal of HBsAg-associated T-cell exhaustion.

However, monotherapy remains insufficient to achieve sustained HBsAg clearance in the majority of patients, largely due to immune exhaustion and immunosuppressive states driven by persistently high antigen burden. In this context, combination therapy has emerged as a major research focus, aiming to restore antiviral immunity through immunomodulatory strategies on the basis of controlled viral replication and reduced antigen load. In recent years, a range of novel therapeutic approaches, including antigen reduction strategies such as small interfering RNA (siRNA) and antisense oligonucleotides (ASO), therapeutic vaccines, immune checkpoint inhibitors, and innate immune agonists, have entered clinical investigation, providing new opportunities for combination therapy (Yin et al., 2023). As illustrated in **Figure 1**, PEG-IFN-α enables the construction of an integrated pathway to functional cure through multi-mechanistic immunological interventions.

## 2 Immunopathogenesis of CHB

The immunological hallmark of CHB is a state of intrahepatic immune suppression and effector cell exhaustion driven by persistently high antigen load, with HBsAg as the central determinant. Serum HBsAg levels are strongly correlated with functional exhaustion of HBV-specific CD8^+^ T cells, characterized by sustained overexpression of inhibitory receptors such as PD-1, TIM-3, and LAG-3, reduced production of effector cytokines including IFN-γ, TNF-α, and IL-2, and impaired cytotoxicity and proliferative capacity. Collectively, these defects markedly diminish the efficiency of infected hepatocyte clearance (Le Bert et al., 2020; Aliabadi et al., 2022; Rehermann, 2024). In parallel, HBsAg and HBeAg directly inhibit dendritic cell (DC) maturation, downregulate the expression of major histocompatibility complex class I/II (MHC-I/II) and costimulatory molecules CD80/CD86, and interfere with innate immune sensing pathways such as TLR9 (Vincent et al., 2011; Tsai et al., 2021), thereby weakening antigen presentation and impairing the priming of naïve T cells (Op den Brouw et al., 2009; Xu et al., 2009; Yonejima et al., 2019).At the level of innate immunity, natural killer (NK) cells exhibit reduced expression of activating receptors, decreased levels of granzyme B and perforin, and compromised cytotoxic activity (Khanam et al., 2021; Yu et al., 2025). Concurrently, regulatory T cells (Tregs) and myeloid-derived suppressor cells (MDSCs) are expanded in both the liver and peripheral blood and suppress effector T-cell and NK-cell function through the secretion of immunosuppressive cytokines such as IL-10 and TGF-β (Pal et al., 2019; Ma et al., 2020; Aliabadi et al., 2022; He et al., 2024). Together, these immune abnormalities establish a stable tolerogenic microenvironment that permits persistent HBV infection and renders spontaneous HBsAg clearance exceedingly rare.

**Figure 2:**
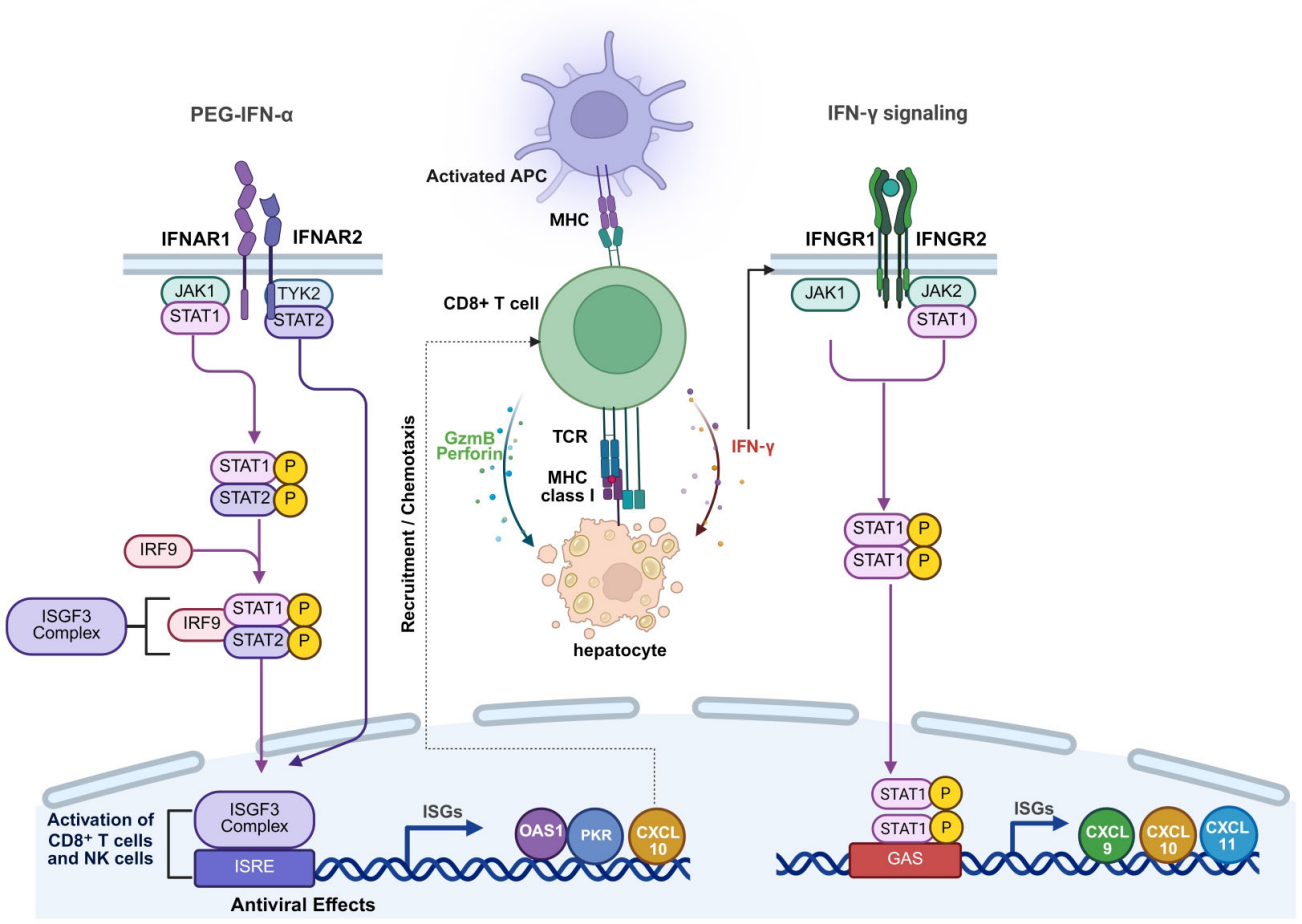
Schematic representation of immune activation mechanisms mediated by PEG-IFN-α and IFN-γ. PEG-IFN-α binds to IFNAR1/IFNAR2, leading to activation of JAK1 and TYK2 and subsequent phosphorylation of STAT1 and STAT2. Together with IRF9, phosphorylated STAT1/STAT2 form the ISGF3 complex, which translocates into the nucleus and binds to interferon-stimulated response elements (ISRE), thereby inducing transcription of interferon-stimulated genes (ISGs), including OAS1, PKR, and CXCL10. These signals enhance antiviral activity and promote activation of CD8^+^ T cells and NK cells. PEG-IFN-α may also enhance APC function, thereby facilitating activation of CD8^+^ T cells. Activated CD8^+^ T cells recognize MHC class I molecules on hepatocytes through the TCR and exert cytotoxic effects through granzyme B and perforin release. IFN-γ binds to IFNGR1/IFNGR2, activating JAK1 and JAK2 and inducing phosphorylation of STAT1 homodimers. These complexes translocate into the nucleus and bind to gamma-activated sequence (GAS) elements, inducing downstream gene expression, including CXCL9, CXCL10, and CXCL11. Together, coordinated activation of type I and type II interferon pathways enhances antiviral signaling, immune cell recruitment, and cytotoxic immune responses.

PEG-IFN-α has the capacity to penetrate this immunosuppressive milieu and promote immune reconstitution, primarily through activation of canonical interferon signaling pathways (**Figure 2**). PEG-IFN-α promotes DC maturation and upregulates the expression of MHC class II and CD40/CD40L, thereby enhancing antigen presentation and T-cell costimulation (Jiang et al., 2024). PEG-IFN-α can promote DC maturation and upregulate the expression of MHC-II and CD40/CD40L, thereby enhancing antigen presentation and T cell co-stimulation. It can also partially restore the effector functions of CD4^+^ and CD8^+^ T cells (Wang et al., 2021; Zhao et al., 2024), increase the secretion of IFN-γ and IL-2, and reduce PD-1–associated inhibitory signaling to a certain extent, while exerting dynamic regulatory effects on inhibitory pathways such as PD-1/PD-L1 during immune reconstitution. Notably, IFN-α has been shown to induce upregulation of PD-L1 expression, suggesting the presence of a negative feedback regulatory mechanism alongside its enhancement of antiviral immunity. In addition, PEG-IFN-α activates NK cells and enhances perforin- and granzyme B–mediated cytotoxicity (Bjorkstrom et al., 2022; Bi et al., 2023). Mechanistically, binding of PEG-IFN-α to interferon-α/β receptor (IFNAR) triggers activation of JAK1 and TYK2, leading to phosphorylation of STAT1 and STAT2, which associate with IRF9 to form the ISGF3 complex. This complex translocates into the nucleus, binds interferon-stimulated response elements (ISREs), and induces transcription of interferon-stimulated genes (ISGs) such as OAS1, PKR, and CXCL10 (Busselaar et al., 2024; Liu et al., 2024), thereby reinforcing the intracellular antiviral state and promoting immune cell recruitment. Meanwhile, IFN-γ signaling through the IFNGR–JAK1/JAK2–STAT1 axis induces the expression of chemokines including CXCL9, CXCL10, and CXCL11, further amplifying the recruitment and effector functions of CD8^+^ T cells and NK cells (Schiefer and Hale, 2024). The coordinated activation of type I and type II interferon pathways constitutes the core molecular basis by which PEG-IFN-α drives immune reconstitution and facilitates HBsAg decline.

## 3 PEG-IFN-α–based combination strategies

NAs constitute the cornerstone of current antiviral therapy and establish a stable virological foundation. However, NA monotherapy alone is insufficient to reverse persistent antigen-driven immune exhaustion, and functional cure rates remain limited. To further improve therapeutic efficacy, combination strategies are being actively explored (Li et al., 2011; Wei et al., 2024). For example, NAs combined with PEG-IFN-α have demonstrated higher rates of HBsAg loss and anti-HBs seroconversion in phase II studies (Bazinet et al., 2020). In addition, toll-like receptor (TLR) agonists (Michelet et al., 2022), therapeutic vaccines (Lian et al., 2022), HBsAg-targeting monoclonal antibodies (Dammacco et al., 2010; Saadoun et al., 2010), and immune checkpoint inhibitors are being investigated in combination with PEG-IFN-α. These approaches aim to overcome persistent immune tolerance through coordinated antigen reduction and immune modulation, thereby improving functional cure rates.

### 3.1 PEG-IFN-α in the context of NA therapy

Under conditions of effective viral suppression achieved by NAs (European Association for the Study of the L, 2025), PEG-IFN-α–based sequential (Lim et al., 2023) or add-on strategies have been widely explored to enhance HBsAg decline and clearance (Rehermann, 2024). Representative clinical studies are summarized in **Table 1**. These approaches aim to improve antiviral immune responses in selected patients and provide a clinical basis for combination therapy strategies.

**Table 1:** Key clinical studies of PEG-IFN-α monotherapy or in sequential/combination therapy with NA in chronic hepatitis B.

Study	Design and population	Treatment regimen	Primary endpoint and HBsAg outcome	Key predictive factors
Ouzan 2013 (Ouzan et al., 2013)	Pre-treated; 10 HBeAg-negative patients; NA ≥3 years, undetectable HBV DNA	Add-on PEG-IFN-α2a (up to 96 weeks)	HBsAg loss in 6/10, 2 developed anti-HBs	Low baseline HBsAg
Bourlière 2017 (Bourliere et al., 2017)	Multicenter Randomized controlled trial(RCT); HBeAg-negative, long-term NA suppression, undetectable HBV DNA	NA monotherapy vs. sequential therapy with NA followed by PEG-IFN-α2a (48 weeks)	At 96 weeks: combo group HBsAg loss 7.8% vs 3.2%	Baseline HBsAg < 1000 IU/mL
PAS Study 2024 (Farag et al., 2024)	Multicenter RCT; HBeAg-negative, NA ≥12 months, HBV DNA <200 IU/mL	PEG-IFN-α2a (48 weeks) add-on vs. NA monotherapy	HBsAg decline ≥1 log: 28% vs 0%; HBsAg loss: 10% vs 0%	HBsAg < 10 IU/mL at week 12; HBsAg < 200 IU/mL
Hu 2018 (Hu et al., 2018)	Multicenter RCT; HBeAg-negative, post-NA treatment, HBV DNA <200 IU/mL	Switch from NA to PEG-IFN-α (48/96 weeks)	HBsAg loss 14.4–20.7%, mostly durable	HBsAg < 1500 IU/mL; HBsAg < 200 IU/mL
Huang 2017 (Liu et al., 2017)	RCT; long-term NA, HBsAg <2000 IU/mL, HBV DNA <20 IU/mL	Switch to PEG-IFN-α2b (60 weeks) vs. continued NA	Only switch group showed HBsAg loss (32.6%)	Low baseline HBsAg
Meta analysis 2024 (Zhang et al., 2024)	7 RCTs, n = 692	Sequential therapy with NA followed by PEG-IFN-α vs. NA monotherapy	HBsAg loss RR ≈ 4.4; serologic conversion RR ≈ 4.0	Low HBsAg, female, ALT elevation, favorable IL28B

In HBeAg-negative patients receiving long-term NA therapy with sustained suppression of HBV DNA, the addition of PEG-IFN-α can further promote HBsAg decline and clearance in a subset of carefully selected patients, although the benefit is not consistent across all individuals. Available evidence suggests that this benefit is mainly observed in patients with lower baseline HBsAg levels. For example, multicenter randomized studies have shown that patients with baseline HBsAg <10 IU/mL are more likely to achieve HBsAg loss, whereas HBsAg >200 IU/mL at week 12 is associated with non-response (Farag et al., 2024).

Early exploratory studies have shown that, in patients with long-term undetectable HBV DNA, addition therapy with PEG-IFN-α can induce HBsAg loss accompanied by anti-HBs seroconversion in a subset of patients (Ouzan et al., 2013; Hu et al., 2018; Zhou et al., 2019). In HBeAg-positive patients with chronic hepatitis B receiving NA therapy, the addition of PEG-IFN-αhas the potential to induce deeper immune responses. Available studies have shown that combination or sequential therapy with PEG-IFN-α can further promote sustained HBsAg decline, and some patients may even achieve HBsAg loss. Current evidence also suggests that lower baseline HBsAg levels and a rapid decline in HBsAg during early treatment are relatively consistent predictors of response. In addition, alanine aminotransferase (ALT) levels, HBV genotype (Lampertico et al., 2018), and certain host immune-related factors may also be associated with treatment outcomes.

Studies of sequential ASO followed by PEG-IFN-α have also shown that (Hanan et al., 2026), in the B-Together phase IIb trial (Buti et al., 2025), some patients who received PEG-IFN-α2a after bepirovirsen treatment maintained HBsAg negativity and sustained HBV DNA suppression at 24 weeks after PEG-IFN-α discontinuation. Patients achieving sustained responses were predominantly those with lower baseline HBsAg levels (≤3000 IU/mL). The study also observed reduced risks of virological or antigenic relapse after treatment discontinuation, although the predefined endpoint for functional cure was not achieved overall.

Overall, multiple phase II clinical studies suggest that combination or sequential use of PEG-IFN-α following antigen reduction may further enhance HBsAg decline and enable relatively higher rates of HBsAg loss in selected patients. Available evidence indicates that the potential benefit of this strategy is mainly concentrated in patients with lower baseline HBsAg levels. In addition, ALT levels, HBV genotype, and certain host immune-related factors may also be associated with treatment outcomes (Lampertico et al., 2018; Yan et al., 2024).Therefore, further optimization of therapeutic strategies combining PEG-IFN-α with antigen-lowering agents in the NA setting remains an important direction toward achieving functional cure.

### 3.2 PEG-IFN-α and therapeutic vaccination

Therapeutic vaccines are designed to restore HBV-specific adaptive immunity by inducing *de novo*, functionally competent antigen-specific T- and B-cell responses. However, their efficacy is often limited under conditions of persistent antigen exposure (Yan et al., 2025). PEG-IFN-α provides a plausible immunological rationale for combination with therapeutic vaccination by enhancing dendritic cell maturation, antigen presentation, and antiviral immune activation, thereby creating a more favorable environment for vaccine-induced responses. Nevertheless, direct clinical evidence supporting PEG-IFN-α–vaccine combination strategies remains limited.

Studies of the therapeutic vaccine GS-4774 have shown that it can enhance HBV-specific T-cell responses; however, no significant reduction in HBsAg levels was observed in patients receiving NA therapy with virological suppression. Early studies also demonstrated its immunogenicity, but without clear antiviral benefit (Lok et al., 2016; Boni et al., 2019). In addition, A phase Ib/IIa study (Ma et al., 2021) conducted in patients receiving NA therapy with virological suppression showed that the therapeutic vaccine BRII-17953, which comprises all three HBV surface envelope proteins (Pre-S1, Pre-S2, and S), can induce HBV-specific humoral and cellular immune responses. Notably, antibody responses were mainly observed in patients who received concomitant short-acting interferon-α (IFN-α), whereas no significant decline in HBsAg was observed overall. In a phase II clinical study (Ji et al., 2025), the combination of the siRNA agent elebsiran for HBsAg reduction with BRII-179 (with some cohorts also receiving IFN-α) was associated with enhanced HBV-specific humoral and cellular immune responses. However, the overall rate of HBsAg clearance remained limited. Furthermore, in a phase II study of PEG-IFN-α combined with elebsiran (Wong et al., 2026), patients who had previously received BRII-179 and developed anti-HBs responses exhibited higher rates of HBsAg loss compared with non-responders. Overall, therapeutic vaccines can enhance HBV-specific immune responses, and PEG-IFN-α may further amplify these effects. However, there is currently insufficient evidence to demonstrate that their direct combination significantly improves sustained HBsAg clearance.

### 3.3 PEG-IFN-α and ICIs

**Figure 3:**
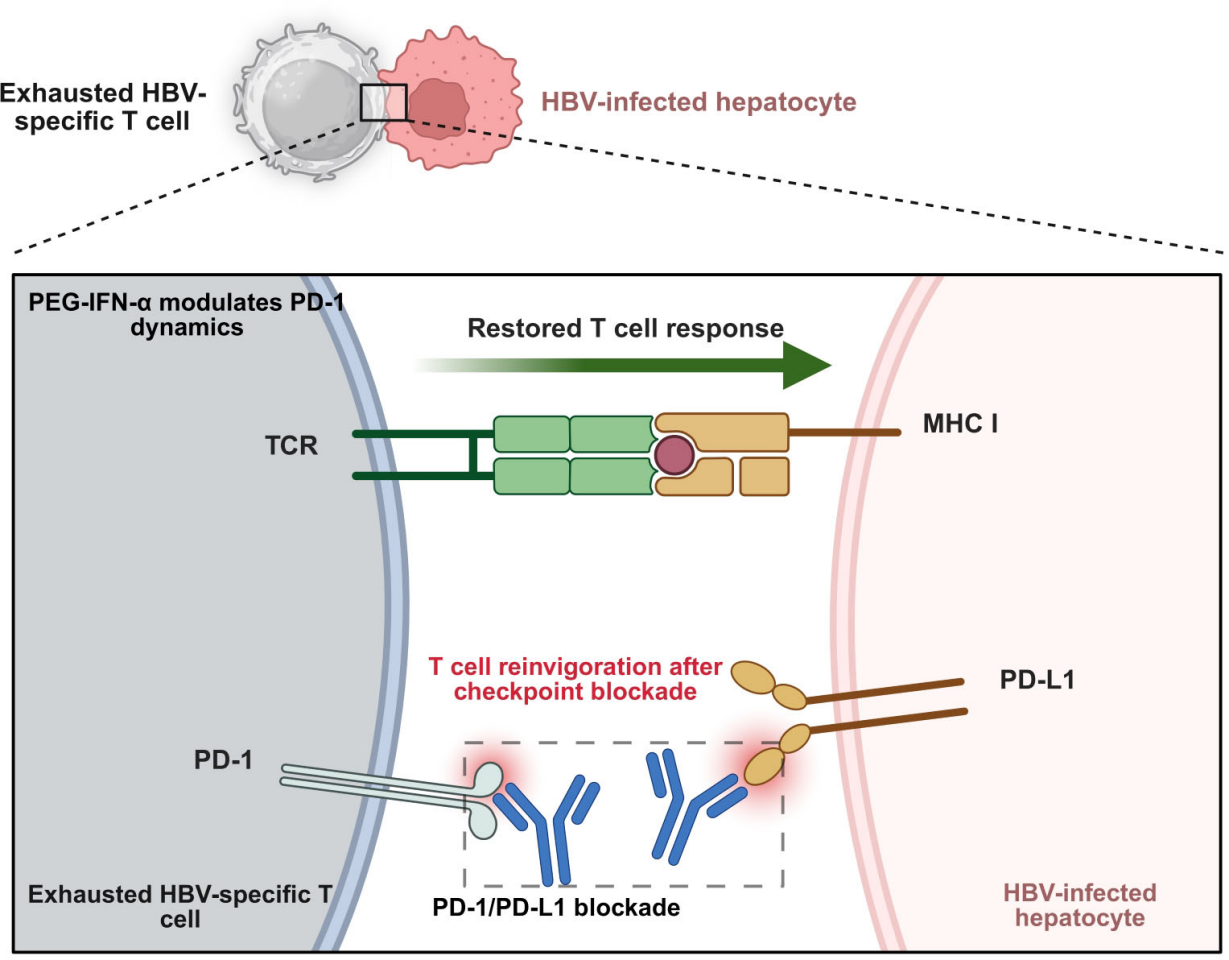
Schematic illustration of T-cell functional restoration mediated by PEG-IFN-α combined with immune checkpoint inhibition. The left side depicts exhausted HBV-specific T cells, whereas the right side represents HBV-infected hepatocytes. PEG-IFN-α may modulate PD-1 expression during immune reconstitution. The upper panel illustrates antigen recognition mediated by interaction between the TCR and MHC-I, corresponding to restoration of T-cell responsiveness. The lower panel depicts the PD-1/PD-L1 inhibitory axis; blockade of this pathway by immune checkpoint inhibitors relieves suppressive signaling and promotes recovery of T-cell effector function. The dashed box indicates the site of checkpoint blockade, and the arrow represents the transition from T-cell exhaustion to reinvigoration.

In CHB, persistent exposure to high viral antigen levels drives HBV-specific T-cell exhaustion, characterized by sustained overexpression of inhibitory receptors such as PD-1, TIM-3, and LAG-3, together with impaired effector function (Wang et al., 2023; Bosch et al., 2024). These findings provide a strong rationale for immune checkpoint blockade as a strategy to restore antiviral immunity. Multiple *in vitro* and translational studies have shown that PD-1/PD-L1 blockade can enhance proliferation and IFN-γ production of HBV-specific CD8^+^ T cells, with particularly pronounced effects in intrahepatic lymphocytes (Li et al., 2020; Liu et al., 2020; Huang et al., 2023) (**Figure 3**).

PEG-IFN-α may improve the efficacy of ICIs by promoting partial immune restoration prior to checkpoint inhibition. Mechanistically, PEG-IFN-α enhances dendritic cell maturation and antigen presentation, upregulates MHC class I and costimulatory molecules, and promotes NK and T-cell activation (Nishio et al., 2021). In addition, interferon signaling may modulate PD-1/PD-L1 axis dynamics and increase the responsiveness of exhausted T cells to checkpoint blockade (Zhou et al., 2025). Recent studies (He et al., 2025) in virologically suppressed CHB patients receiving PEG-IFN-α add-on therapy further demonstrated recovery of HBV-specific T-cell responses after treatment, particularly among those with lower baseline HBcrAg levels, supporting the immune-priming role of PEG-IFN-α prior to ICI therapy.

Current clinical evidence for ICIs in CHB remains limited and is mainly derived from early-phase studies. A phase I study of nivolumab (Gane et al., 2019) showed that some patients receiving NA therapy with virological suppression experienced declines in HBsAg levels; however, sustained HBsAg loss remained uncommon. Early studies of PD-L1–targeting antibodies also suggested acceptable safety profiles and measurable immunological changes, but definitive evidence for durable HBsAg clearance is still lacking.

At present, direct clinical evidence for the combination of PEG-IFN-α and ICIs in CHB remains scarce, and most available experience is extrapolated from HBV-related hepatocellular carcinoma cohorts68. Immune-mediated hepatitis flares, ALT elevations, and the potential risk of HBV reactivation also warrant careful monitoring. Therefore, PEG-IFN-α plus ICI therapy in CHB should currently be regarded as an exploratory strategy requiring validation in prospective clinical trials.

### 3.4 PEG-IFN-α and HBsAg-targeting monoclonal antibodies

HBsAg-targeting neutralizing monoclonal antibodies can rapidly reduce antigen exposure by binding circulating HBsAg and facilitating its clearance (Vincenzetti et al., 2026). Preclinical studies have shown that these antibodies not only decrease serum HBsAg levels and SVPs, but may also partially reverse the immunosuppressive state caused by persistent antigen exposure, thereby creating favorable conditions for subsequent immune reconstitution.

However, HBsAg monoclonal antibody monotherapy is generally insufficient to fully restore HBV-specific immune function, and its long-term ability to achieve functional cure remains limited. Previous preclinical studies have shown that engineered neutralizing antibodies targeting the preS1 or S regions can markedly reduce HBsAg levels and enhance intrahepatic HBV-specific T-cell responses (Zhang et al., 2016; Golsaz-Shirazi et al., 2017; Wi et al., 2017; Hong et al., 2021; Beretta and Mouquet, 2022; Gehring and Salimzadeh, 2024).

At present, direct clinical evidence for the combination of PEG-IFN-α with HBsAg-targeting monoclonal antibodies remains limited. Recent early-phase studies have mainly focused on HBsAg-neutralizing antibodies represented by VIR-3434 (tobevibart) (Lempp et al., 2023), often evaluated in combination with the siRNA agent VIR-2218 (elebsiran), with treatment arms including regimens with or without PEG-IFN-α. Available studies suggest that, on the basis of marked antigen reduction, the addition of PEG-IFN-α may further enhance immunomodulatory effects. however, its independent contribution to sustained HBsAg clearance has not yet been clearly defined.

Overall, the combination strategy of PEG-IFN-α with HBsAg-targeting monoclonal antibodies remains at an early exploratory stage (Lempp et al., 2023). Larger clinical studies are still needed to clarify its value in achieving functional cure.

### 3.5 PEG-IFN-α and innate immune agonists

Innate immune agonists targeting the TLR-7, TLR-8, and STING pathways may provide a rational partner for PEG-IFN-α by activating upstream antiviral sensing pathways and amplifying type I interferon responses. Through activation of dendritic cells and monocytes/macrophages, these agents induce endogenous interferons and a broad range of ISGs, thereby enhancing innate immune activity and facilitating subsequent adaptive immune responses. Such mechanisms make them attractive immunomodulatory candidates in chronic HBV infection.

Representative studies have shown that the TLR-7 agonist GS-9620 can induce IFN-α responses, reduce HBV RNA levels, partially lower HBsAg, and enhance intrahepatic CD8^+^ T-cell activity (Gane et al., 2015; Boni et al., 2018; Janssen et al., 2018; Mori et al., 2023). Similarly, the TLR-8 agonist selgantolimod (GS-9688) has demonstrated the ability *in vitro* and in early clinical studies to activate monocytes and dendritic cells and to augment HBV-specific T-cell responses (Amin et al., 2021; Ayithan et al., 2021; Gane et al., 2023; Janssen et al., 2024). However, in patients with CHB, the antiviral effects of these agents as monotherapy, particularly with respect to sustained HBsAg reduction, have generally been modest.

PEG-IFN-α promotes antiviral immune recovery through the IFNAR–JAK–STAT pathway by inducing ISGs, enhancing antigen presentation, and strengthening NK-cell and HBV-specific T-cell function. Therefore, combining PEG-IFN-α with innate immune agonists may theoretically generate complementary or synergistic effects through simultaneous activation of endogenous innate sensing pathways and exogenous interferon signaling.

At the same time, overlap in immune activation pathways may increase the risk of additive toxicity. Available clinical data (Agarwal et al., 2018; Janssen et al., 2018) suggest that TLR-7/8 agonists are generally well tolerated in CHB, with most adverse events limited to mild-to-moderate influenza-like symptoms and transient systemic immune activation, partially overlapping with the known safety profile of interferon therapy (Janssen et al., 2018).

To date, no systematic clinical trials have directly evaluated PEG-IFN-α in combination with TLR or STING agonists in CHB, and current evidence is largely derived from mechanistic studies and preclinical models. Consequently, this strategy remains at the proof-of-concept and early exploratory stage. Future studies are needed to define its true synergistic potential, optimal therapeutic window, and the patient populations most likely to benefit.

## 4 Challenges, biomarkers, and patient selection

Although multiple PEG-IFN-α–based combination strategies have shown potential to promote HBsAg decline and improve serological outcomes (Deng et al., 2025), their clinical benefit remains largely confined to a subset of patients, and overall functional cure rates still require improvement (Peng et al., 2024). Identifying the patients most likely to benefit, optimizing treatment timing, and enhancing response rates have therefore become central challenges in current clinical translation. The major obstacles and potential optimization strategies are summarized in **Table 2**.

**Table 2:** Key challenges and optimization strategies for PEG-IFN-α–based combination immunotherapy.

Challenge category	Specific issues	Optimization strategies	Reference
Safety	Flu-like symptoms, bone marrow suppression, ALT elevation; increased immune-related risks when combined with other immunotherapies.	Careful patient selection (e.g., preserved liver function, absence of active autoimmune disease), close monitoring, and individualized dosing.	(van Zonneveld et al., 2005; Bourliere et al., 2017; Farag et al., 2024; Wang et al., 2024b; Zhang et al., 2024)
Tolerability/Adherence	Long treatment duration, frequent adverse effects, suboptimal injection adherence.	Pre-treatment education, reinforced follow-up support, consideration of phased or shorter regimens.	(Bourliere et al., 2017; Hu et al., 2018; Zhang et al., 2024)
Efficacy Heterogeneity	Low monotherapy response rate, significant interindividual variability, lack of efficacy in some patients.	Stratification based on biomarkers such as baseline HBsAg, HBV RNA, and IL28B genotype.	(Bourliere et al., 2017; Brakenhoff et al., 2022; Chu et al., 2022; Farag et al., 2024)
High Antigen Burden	Persistent high HBsAg levels promote immune tolerance and limit immunologic response.	Initiate antigen-lowering strategies (e.g., siRNA, ASO, monoclonal antibodies) before introducing PEG-IFN-α to enhance immunomodulatory efficacy.	(Yuen et al., 2021; Shechter et al., 2025)
Patient Heterogeneity	Host factors such as IL28B genotype, sex, age, and baseline ALT influence treatment outcomes.	Develop multidimensional predictive models and implement precision stratification and individualized therapeutic planning.	(Bourliere et al., 2017; Xu et al., 2024; Zhang et al., 2024)

First, persistent viral antigen exposure remains a major barrier to treatment efficacy (Taddese et al., 2025). Even after virological suppression is achieved with NA therapy, cccDNA and integrated HBV DNA may continue to produce HBsAg, making immune tolerance difficult to fully reverse. In addition, inter-individual differences in the degree of HBV-specific T-cell exhaustion, intrahepatic inflammatory activity, and host genetic background further contribute to the heterogeneity of treatment response to PEG-IFN-α–based combination therapy.

With regard to biomarkers, baseline HBsAg level remains one of the most widely used predictors (Tseng et al., 2025), particularly in HBeAg-negative patients receiving NA therapy with virological suppression. However, reliance on HBsAg alone has limitations. Combining HBsAg with other markers, such as HBV RNA (Testoni et al., 2024) and HBcrAg, may provide a more comprehensive reflection of residual viral activity and thereby improve predictive accuracy. Recent studies (Vecchi et al., 2024) have further shown that in HBeAg-negative patients receiving long-term NA therapy with virological suppression, lower baseline HBcrAg levels were associated with greater HBsAg decline and better recovery of HBV-specific T-cell function after PEG-IFN-α add-on therapy. These findings suggest that HBcrAg may not only reflect residual viral activity, but also serve as a practical biomarker for identifying patients more likely to benefit from PEG-IFN-α.

Immunological biomarkers may also help refine patient selection. Chemokines such as IP-10 (Wang et al., 2024) and CXCL10 (Yang et al., 2024) may reflect activation of interferon signaling pathways and have shown potential as early predictors of treatment response in some studies. In addition, the functional status of HBV-specific T cells, as well as the expression of exhaustion-associated molecules such as PD-1, TIM-3 (Yu et al., 2021), and LAG-3, may help identify patients more suitable for immunomodulatory therapy.

Beyond molecular biomarkers, clinical factors should also be incorporated into a comprehensive assessment. HBV genotype (Guo et al., 2019), HBeAg status (Guo et al., 2023), ALT levels (Wang et al., 2025), age, degree of liver fibrosis, and baseline liver function may all influence treatment outcomes. Overall, patients with stable virological suppression, lower HBsAg levels, preserved liver function, and good treatment tolerability may represent more favorable candidates for PEG-IFN-α combination therapy.

On the other hand, the safety profile of PEG-IFN-α should not be overlooked. In addition to influenza-like symptoms, bone marrow suppression, and fatigue (Wang et al., 2024a), thyroid dysfunction as well as neuropsychiatric adverse events such as insomnia, anxiety, and mood changes may also limit its clinical use. Therefore, potential benefits must be carefully balanced against treatment-related risks when pursuing functional cure.

**Figure 4:**
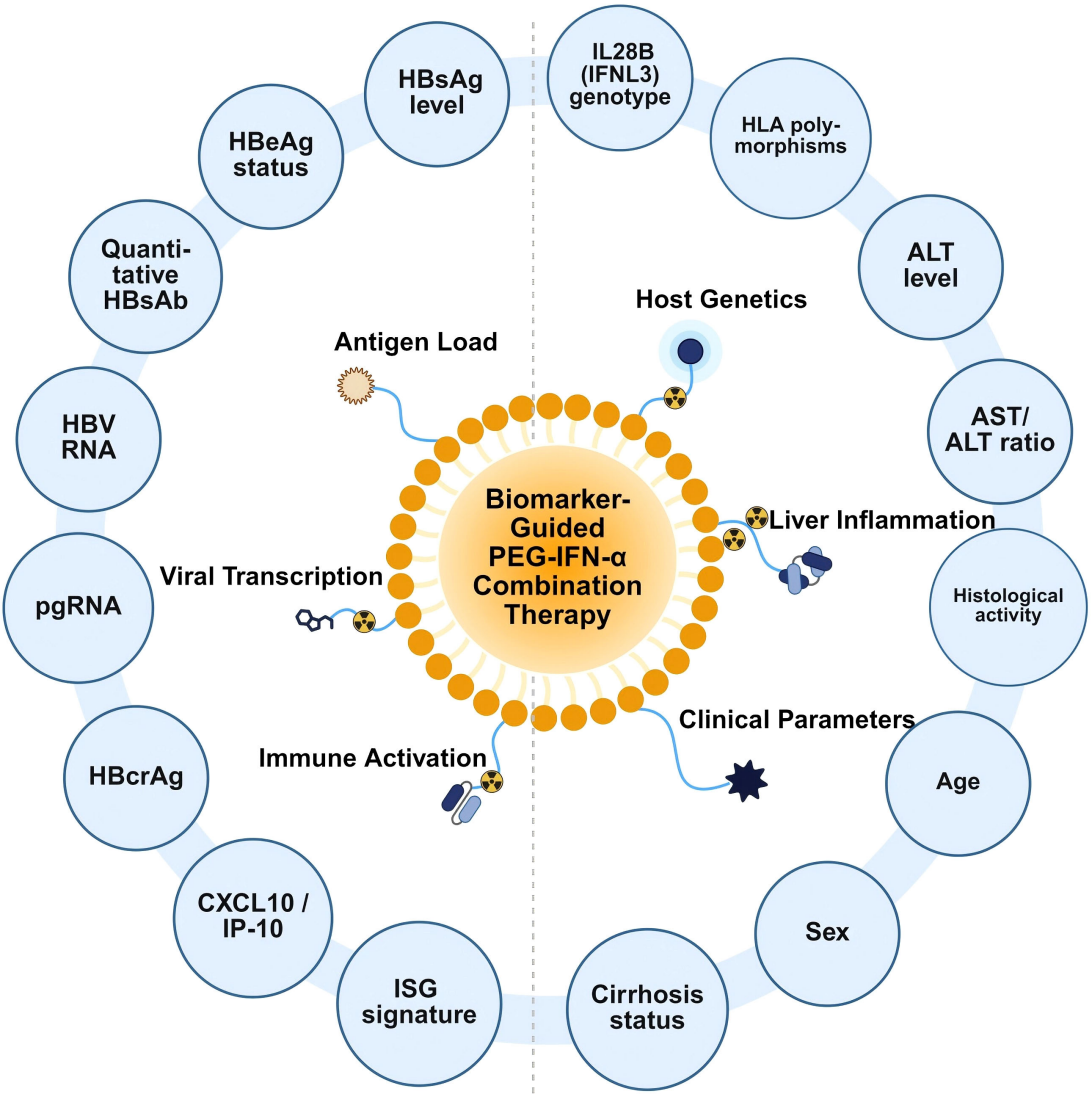
Potential biomarkers for guiding PEG-IFN-α–based combination therapy. A schematic illustration of potential biomarkers to guide patient selection for PEG-IFN-α–based combination immunotherapy. The inner ring categorizes biomarkers into six main biological domains: antigen load, viral transcription, immune activation, host genetics, liver inflammation, and clinical parameters. The outer ring displays representative biomarkers within each domain, such as HBsAg level, pgRNA, CXCL10/IP-10, IL28B genotype, ALT level, and cirrhosis status. This multi-dimensional classification highlights the importance of integrated virological, immunological, and host factors for precision therapy in chronic HBV infection.

Future research should move beyond a uniform treatment approach toward precision stratified therapy, integrating baseline virological features, host immune status, and dynamic on-treatment biomarker changes to guide individualized combination strategies (**Figure 4**). Through optimization of patient selection, treatment sequencing, and duration, the clinical value of PEG-IFN-α–based combination strategies for functional cure of chronic hepatitis B may be further improve.

## 5 Conclusions and future perspectives

Although NAs can achieve long-term and stable suppression of HBV replication, the rate of functional cure in patients with chronic hepatitis B remains low because of their limited effects on cccDNA activity and HBsAg production. As the only currently approved therapy capable of inducing HBsAg loss, PEG-IFN-α continues to play an irreplaceable role in CHB treatment through its direct antiviral activity and immunomodulatory effects.

With the growing understanding of HBV immunopathogenesis, PEG-IFN-α is no longer confined to the traditional monotherapy model, but has gradually become an important component of combination therapy (Wong et al., 2025). Combination strategies involving PEG-IFN-α with antigen-lowering agents, therapeutic vaccines, immune checkpoint inhibitors, and innate immune modulators are currently under active investigation.

Looking forward, improving functional cure rates will depend on more precise patient selection, better optimization of treatment timing and duration, and well-designed prospective clinical studies that balance efficacy and safety. With continued advances in immunotherapy and precision medicine, PEG-IFN-α–based combination strategies may enable functional cure to benefit a broader population of patients with chronic hepatitis B.
